# On the importance of modeling balloon folding, pleating, and stent crimping: An FE study comparing experimental inflation tests

**DOI:** 10.1002/cnm.3249

**Published:** 2019-12-23

**Authors:** Markus A. Geith, Krzysztof Swidergal, Bernd Hochholdinger, Thomas G. Schratzenstaller, Marcus Wagner, Gerhard A. Holzapfel

**Affiliations:** ^1^ Institute of Biomechanics Graz University of Technology Graz Austria; ^2^ Biomedical Engineering Department King's College London United Kingdom; ^3^ Faculty of Mechanical Engineering Ostbayerische Technische Hochschule Regensburg Germany; ^4^ DYNAmore Swiss GmbH Switzerland; ^5^ Department of Structural Engineering Norwegian University of Science and Technology Trondheim Norway

**Keywords:** catheter, coronary, crimping, finite element, numerical, stent

## Abstract

Finite element (FE)–based studies of preoperative processes such as folding, pleating, and stent crimping with a comparison with experimental inflation tests are not yet available. Therefore, a novel workflow is presented in which residual stresses of balloon folding and pleating, as well as stent crimping, and the geometries of all contact partners were ultimately implemented in an FE code to simulate stent expansion by using an implicit solver. The numerical results demonstrate that the incorporation of residual stresses and strains experienced during the production step significantly increased the accuracy of the subsequent simulations, especially of the stent expansion model. During the preoperative processes, stresses inside the membrane and the stent material also reached a rather high level. Hence, there can be no presumption that balloon catheters or stents are undamaged before the actual surgery. The implementation of the realistic geometry, in particular the balloon tapers, and the blades of the process devices improved the simulation of the expansion mechanisms, such as dogboning, concave bending, or overexpansion of stent cells. This study shows that implicit solvers are able to precisely simulate the mentioned preoperative processes and the stent expansion procedure without a preceding manipulation of the simulation time or physical mass.

## Graphical abstract

This study shows that finite element analyses of coronary stent implantations can be significantly improved by adopting geometries as well as residual stresses and strains from preoperative processes such as folding, pleating and crimping. During the mentioned processes, stresses inside the balloon catheter membrane and stent material reached a severe level. Furthermore, this study showed that up‐to‐date implicit solvers are able to precisely analyze preoperative processes and coronary stent implantations without manipulating the simulation time or the physical mass.

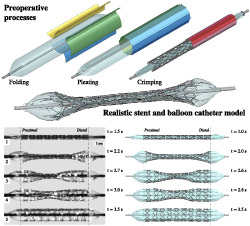

## 1 INTRODUCTION

The number of coronary stent implantations (CSIs) rises rapidly, particularly in industrialized countries such as Austria, where the number of implanted stents increased by 21% between 2012 and 2017.^1^ The most challenging complication after CSI is the adverse event of restenosis, which occurs at rates as high as 20% for bare metal (BMS) and drug‐eluting stents (DES) despite improvements.^2^ To reduce the severity of neointimal hyperplasia due to excessive cell proliferation and migration after the use of BMS,^3^ researchers around the world are undertaking great efforts to apply cell growth–inhibiting drugs onto the stent surface, eg, sirolimus^4^ and paclitaxel.^5^ This resulted in the first generation of DES, which initially showed good results in lowering the rate of early restenosis from 30%^6^, ^7^ to about 10%.^8^, ^9^, ^10^, ^11^ However, first clinical long‐term outcomes revealed that patients having the first‐generation DES implanted tend to have a higher risk of late vascular events (greater than 3 weeks, less than 1 year) compared with patients with BMS. The reason is a delayed endothelialization of the stent struts, which entails the danger of late in‐stent thrombosis^12^, ^13^ and incomplete embedding that again causes malapposition.^14^

In order to regulate and to extend the delivery of drugs after the stent is implanted, it is currently common to apply polymers to the stent surface as a carrier for pharmaceutical substances. Such stents represent the second generation of DES. Some of these polymers seem to be ineligible because of possible inflammatory responses.^15^, ^16^ Lately, clinical studies have confirmed that even the newer generation of DES are not superior to BMS and show equal fatality rates.^2^, ^17^, ^18^ The study of Timmins et al^19^ prove that increasing arterial wall stress leads to a more severe neointimal response in the form of cell migration and proliferation. Therefore, to reduce the fatality rate of BMS as well as DES for CSI, scientists and manufacturers need to develop safer stents by significantly lowering the risk of vascular injuries. This goal may be achieved by reducing the load on the artery applied by the stent and the balloon catheter. Therefore, engineers need to change the stent/balloon geometry, their material, or both.

Stents for CSI are usually expanded by the inflation of a noncompliant balloon catheter. For minimally invasive and endovascular treatments, balloon catheters consist mainly of a blow‐molded, cylindrical balloon with short tapers at both ends, which are adhered to a catheter. Stents and balloon catheters pass through three important preoperative processes before they are—purely mechanically—ready to be implanted as a unit: (a) in the folding process, three wings of the balloon are formed with curved folding blades; (b) during the pleating process, the balloon is wrapped around the catheter, creating a distorted star‐shaped balloon cross‐section; (c) in the crimping process, the stent is plastically deformed by reducing its diameter, and it is pressed onto the pleated balloon. The load acting on the balloon and stent during thefolding, pleating, and crimping processes cannot be measured. The movement of the displacement‐controlled blades of all these processes is based on empirical values only. The authors assume that wrong process parameters can adversely increase initial residual stresses inside the balloon catheter and the stent before CSI. The change in the residual stresses could negatively influence typical expansion mechanisms and the final geometry of the implanted stent. This circumstance could result in severe damage to the balloon catheter, the stent, and, of course, the arterial wall. Because of this fact, it seems even more critical for the optimization process of balloon catheters and stents to know and to consider initial residual stresses and strains experienced during the preoperative processes

Finite element analysis (FEA) has become an effective tool not only to investigate the expansion behavior of balloon catheters and stents. FEA can save valuable development time and costs by replacing or reducing extensive material tests and ethically questionable animal experiments. FEA can also provide information about whether the strength of the stent is sufficient to withstand the stresses during a CSI.

Especially the expansion behavior of a stent depends decisively on the balloon catheter type, ie, its geometry, the applied folding and pleating technique, and its operating pressure. In first FEA, pressure was applied to the luminal surface of the stent to expand it.^20^, ^21^, ^22^, ^23^, ^24^, ^25^ These early studies demonstrated already the great potential of FEA in analyzing the stresses and strains of an expanding stent. However, it is becoming more apparent that it is essential to integrate an important contact partner of the stent, the balloon catheter, into up‐to‐date FEA. Therefore, during the last two decades, FEA have been performed with balloon catheter models, showing an increasing level of geometrical detail. The first refinement step includes FEA with simple rigid cylindrical shells.^26^, ^27^, ^28^, ^29^ By increasing the diameter of these cylindrical models, a displacement‐controlled expansion of the stent could be simulated.

On the contrary, using a simple cylinder for stent expansion during the FEA does not allow to mimic a realistic contact pressure distribution between stent and balloon. Ju et al^30^ and Kiousis et al^31^ overcame this problem by pressurizing the luminal surface of elastic cylinders with a hyperelastic material model, which constitutes the second refinement step. Nevertheless, the influence of the unsymmetrical star‐like shape of a folded balloon on the expansion process of the stent cannot be investigated with such models. It is documented that the unsymmetrical folding of the balloon catheter has a more considerable influence on the expansion behavior of a stent than the mechanism of the stent itself.^32^ By implementing a hyperelastic balloon model consisting of a folded model with open ends and star‐shaped cross sections, De Beule et al^33^ initiated the third refinement step.

Open balloon models may not be used to study the influence of tapers at both ends of a balloon catheter on stent expansions. Without the tapers, hyperelastic balloon models lack a design‐based resistance to an unimpeded expansion of the balloon ends. Therefore, the fourth refinement stage characterizes modern FEA of stent implantations in which folded balloon models with tapers are implemented. In order to gain the detailed geometry of a folded balloon model, different approaches have been pursued, which include (a) the modeling of the balloon membrane in an already pleated and folded state by using computer‐aided design (CAD) methods or by the application of mathematical transformation techniques,^34^, ^35^, ^36^, ^37^ (b) the extraction of the balloon geometry using data from 3D micro‐computed tomography (micro‐CT) scans,^38^ and (c) the application of pressure to the outer surface of the membrane to deflate the balloon.^39^, ^40^ However, to the authors' knowledge, no study has yet considered the modeling and simulation of the folding and pleating processes mentioned above by implementing the one‐to‐one blade geometry of a multi‐head tool in order to recreate a realistically folded balloon model. This further refinement step is necessary because the possibility that balloon membranes are exposed to high stresses during these preoperative processes cannot be excluded. High stresses can lead to plastic strains or even damage to the membrane material, which can significantly influence the expansion mechanism of the balloon catheter and, therefore, also of the stent.

A few other groups such as Wu et al^41^ and Li et al^42^ reach a broader perspective on known issues of stent optimization through the additional consideration of the crimping process in their FEA. For both the expansion and the crimping process, they use radially expandable rigid cylinders to vary the diameter of the stent. Schiavone et al^43^ simulated the crimping procedure following the iris principal, where twelve rigid blades of a multihead tool press the stent on a linear elastic isotropic balloon model. The results show that the tested stent is already exposed to a significant amount of stress during crimping and thence substantiated the assumption that damage to stents may arise even before their implantation. This matches with the results from Möller et al^44^ which reported about significant residual stresses in crimped stents after using the X‐ray diffraction method. It is also essential to consider an investigation of balloon catheters during the crimping process. Rondeau et al^45^ demonstrated in their study that balloon membranes show alterations on their surface like streaks and even abrasion after stents were crimped on them.

CSI is a quasi‐static procedure. Nevertheless, almost all mentioned research groups used explicit solvers for the FEA of CSI. On the one hand, convergence problems due to nonlinearities, large deformations, sliding contact, and coarse meshes are usually prevented. On the other hand, the explicit time integration method allows only small time steps, which can culminate in several days of computational time.^46^ A drastic reduction of the FEA simulation time of CSI from several seconds to fractions of a second and a mass scaling are popular but possibly mistrustful countermeasures. Although the method of mass scaling^47^ allows larger time steps, it can lead to an additional falsification of the results in CSI simulations in which large deformations and dynamic effects are expected. Despite increased computational costs and high demands on the mesh quality, contact definition, and boundary conditions, the implicit time integration method allows larger time steps without adding nonphysical mass or changing the quasi‐static character of the CSI simulation. Bukala et al^37^ have been the first group to demonstrate the benefits of an implicit solver by analyzing the crimping and stent expansion process. However, no FEA‐based studies are known in which all preoperative processes (folding, pleating, and stent crimping) were simulated neither with an implicit nor an explicit solver.

For this reason, this study aims to show how existing FEA simulations of CSI can be supplemented by considering the folding, pleating, and crimping procedures and by using an implicit solver. Therefore, FEA of the three preoperative processes were performed following an optimized computer‐aided engineering (CAE) process workflow: starting with designing the geometries of a balloon catheter and a stent, going through discretizing the 3D‐models with a preprocessor, continuing with simulating the processes with an FEA program using the highly precise implicit time integration method, and ending with the evaluation of the results in a postprocessor. Finally, initial residual stresses and all generated geometries were used in a final simulation of an expanding stent to investigate the influence of the preoperative processes. The results of the FEA were verified with images from micro‐CT scans and with an inflation test documented with a high‐speed camera. And, finally, the FEA using the new method was compared with simulations following (more) classical approaches known from the literature. Along with the new approach devised by Geith et al^48^ the authors of this study mainly focus on the quantification of vascular damage due to CSI by analyzing biomechanical and structural changes of coronary arteries to define a more precise damage and growth model for coronary arteries. The present study suggests important preliminary steps for subsequent numerical analyses.

## 2 METHODS

### 2.1 Balloon catheter and stent

**Figure 1:**
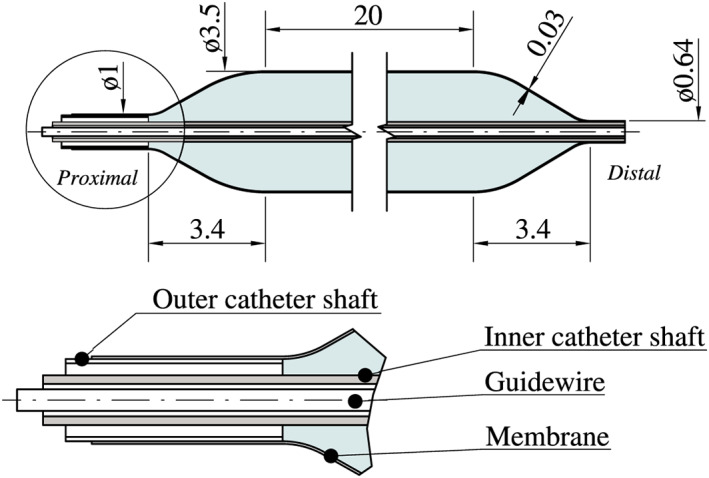
Baroonda stent delivery system (SDS) balloon catheter. While the distal end of the membrane is fused to the inner catheter shaft, the proximal end, connected to the outer catheter shaft, is free to slide along its longitudinal axis. All dimensions are in millimeters and taken from own measurements

**Figure 2:**
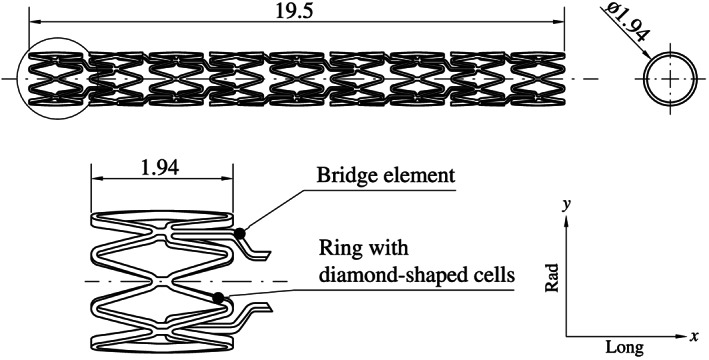
Half‐section of the ESPRIT V1 stent before the preoperative processes. The stent has nine rings along its longitudinal axis with respectively eight diamond‐shaped cells. The rings are connected to each other with two bridge elements. The structure of the stent was designed as symmetrically as possible to reduce twists and asymmetrical expansion/elongation. All dimensions are in millimeters and taken from own measurements

For this study, the over‐the‐wire balloon catheter Baroonda stent delivery system (SDS, 08BO‐3520A, Bavaria Medizin Technologie GmbH, Wessling, Germany) with a tri‐folded and noncompliant balloon membrane made from the thermoplastic Grilamid L25 (EMS‐Chemie AG, Domat/Ems, Switzerland) was used (Figure 1). Once the operating pressure of *p*=1.0 MPa for the Baroonda is applied and the membrane is fully inflated, the original state of the balloon cannot be restored, and the catheter needs to be disposed. As mentioned, balloon catheters for coronary stents are usually blow molded. Therefore, it can be assumed that the polymer chains of the Grilamid L25 align in the circumferential direction of the balloon, and thus strongly anisotropic material properties prevail. Because of the lack of experimental data in the literature, only material parameters of unprocessed, isotropic Grilamid were available to the authors. In addition, a stent made of 316 LVM stainless steel (ThyssenKrupp Steel Europe, Duisburg, Germany), stress‐relieved stainless steel, with closed cells, based on the prototype ESPRIT V1 design, was investigated (Figure 2). The ESPRIT V1 design was developed during a separate preliminary study. Table 1 presents the essential material parameters for Grilamid L25 and 316LVM.

**Table 1:** Material properties of the raw materials for the Baroonda SDS balloon catheter (Grilamid L25, unprocessed, isotropic – PA 12) and the ESPRIT stent (316LVM – 1.4441) provided by the manufacturers

		Grilamid L25 (cond., unprocessed, isotropic)	316 LVM
Density *ρ*	ton/mm^3^	1.010E‐09	7.850E−09
Young modulus *E*	MPa	1100	2.100E+05
Yield point *R*_p0.2_	MPa	–	332
Tensile strength *R*_m_	MPa	45	671
Poisson ratio *v*		0.40	0.29

SDS, stent delivery system.

The distal taper of the balloon membrane is connected to the inner catheter shaft, which exits the balloon at the proximal end. The proximal taper is connected to the outer catheter shaft, which can slide freely to a certain degree along its longitudinal axis in the global *x*‐direction, see Figure 2. Thus, the balloon catheter can compensate elongations during the preoperative processes and the stent expansion. On the basis of the dimensions measured with the light microscope VHX‐2000 (Keyence, Osaka, Japan), a 3D design of the unfolded and untreated balloon catheter was created with Autodesk Inventor 2018 (Autodesk, San Rafael, USA). Figures 1 and 2 show all necessary dimensions of the Baroonda SDS balloon catheter and the ESPRIT V1 stent. Using a 2D template, which was wrapped around a cylinder and extruding with the “emboss” feature in Inventor, an exact 3D model of the stent could be created. Finally, the ESPRIT V1 stent was manufactured by MeKo Laser Material Processing GmbH (Sarstedt, Germany) by laser cutting, annealing, and electropolishing. The finished ESPRIT V1 stent has a total stent length of 19.5 mm, an outer diameter of 1.94 mm, a strut width of about 80 μm and a depth of 120 μm.

### 2.2 Preoperative processes

**Figure 3:**
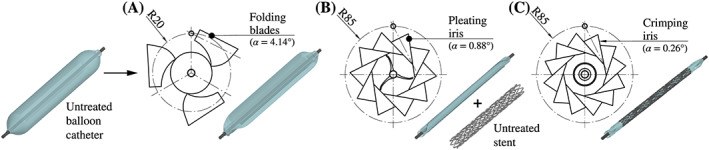
Overview of the three preoperative processes. In the folding process A, the untreated balloon catheter is pressed into a star‐like shape with three wings; during pleating B, the wings are folded over and pressed against the inner catheter shaft; and, finally, in the crimping process C, the stent is crimped onto the folded balloon catheter. The blades rotate with the displacement angle *α* around the pivot axes, which are parallel to the longitudinal direction of the balloon/stent

To ensure that stents and arteries are not getting overstretched during CSI, balloon membranes are made from rather noncompliant materials. Therefore, the balloon membrane has to be folded and pleated to reduce its diameter until it is small enough to crimp a stent onto it. During the folding process (Figure 3A), the balloon catheter is pressurized with 0.1 MPa while three blades press the cylindrical membrane into the characteristic star shape with three slightly slanted wings within a process time of 2 seconds. This creates a preferred folding direction of the wings. As soon as the star shape is formed, a vacuum of −0.1 MPa is drawn to prevent a springback.

The second process, the so‐called pleating (Figure 3B), serves to press the balloon wings onto the catheter. Ten blades are arranged in the shape of an iris. By rotating the blades, the diameter of the iris can be reduced in 2 seconds. The pleating and folding process was performed with a customized multihead tool (MSI, Flagstaff, USA) by the manufacturer of the balloon catheter. After the three wings are pressed around the catheter tube, a vacuum is again generated.

During the crimping process (Figure 3C), the pleated balloon catheter is placed together with the stent inside the crimper, which features 12 blades. By reducing the iris diameter, the stent is plastically deformed and pressed onto the pleated balloon. After a process time of 2 seconds, the stent is compressed to its smallest diameter. Once the iris opens again and crimp pressure is reduced, the stent recoils to its final diameter. For the crimping procedure, a fully functional and automated prototype was used. After a sterilization procedure, the balloon catheter with the stent is ready for CSI.

### 2.3 Inflation tests and micro‐CT scans

**Figure 4:**
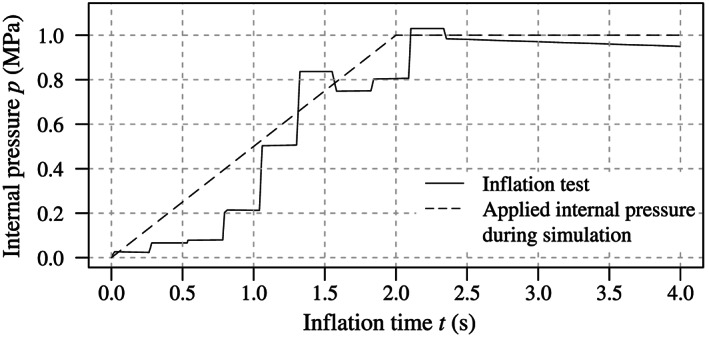
Internal pressure *p* of the balloon catheter expanding the stent vs time *t* during an inflation test. While the realistic pressure curve shows rapid increases followed by plateaus, a linear pressure increase of 2 seconds was assumed for the finite element analysis (FEA) of coronary stent implantation (CSI)

To validate the numerical results of the present work, water was pumped into the balloon catheter to expand the stent. For the nominal diameter of 3.5 mm, an internal pressure of *p*=1.0  MPa was deployed within *t*∼2 seconds with the commercial pump BasixCOMPAK (Merit Medical, South Jordan, USA) with a handwheel. The inflation finished after another 2 seconds. In parallel, the pressure was measured at the adapter of the balloon catheter with the sensor XMLP600PP13FQ3 (Telemecanique, Rueil‐Malmaison, France). The pressure curve usually varies greatly with every trial and shows plateaus and sudden increases, as presented in Figure 4. This is due to the manually operated pump, air pockets inside the catheter shaft, the viscous flow of the contrast medium solution, and the abrupt volume expansion when a certain pressure is reached and the stent bursts open. During the inflation test, the balloon catheter was filmed with the high‐speed camera HCC‐1000 (VDS Vosskühler, Osnabrück, Germany) to measure the diameter of the stent at its ends and in its center. In addition, to analyze its cross‐section, the Baroonda SDS balloon catheter was scanned with the X‐ray microtomography scanner (micro‐CT) GEv|tome|xs (General Electric, Boston, USA).

### 2.4 Numerical simulations

**Figure 5:**
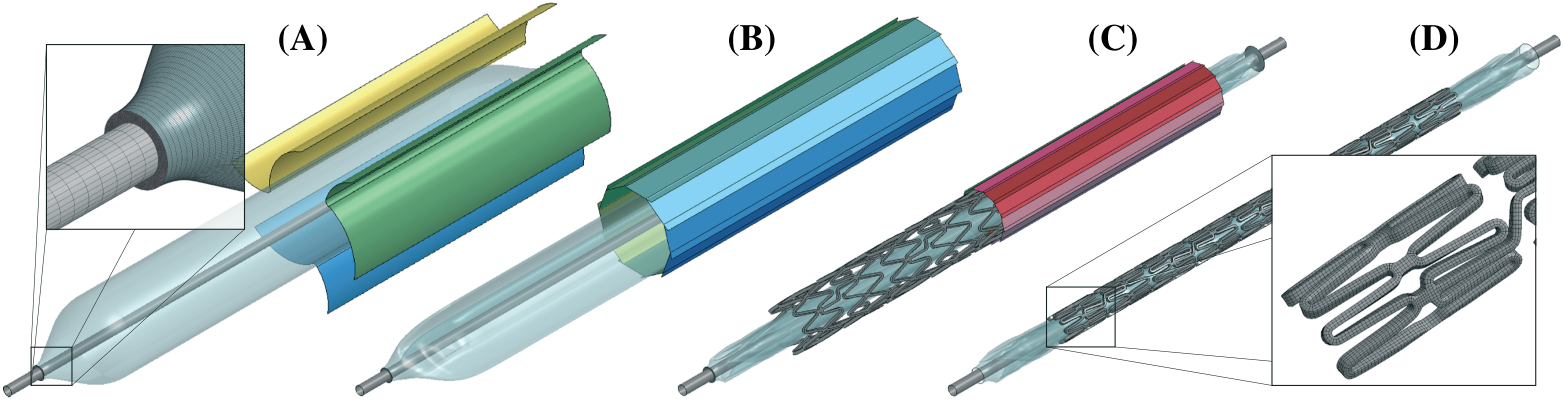
Reduced geometries for the finite element analysis (FEA) of the folding A, pleating B, crimping C, and expansion process D. For better visualization, only one‐half of the blades of the devices is visible

For the FEA, the mid‐surface of the 3D‐CAD model of the Baroonda SDS balloon membrane was extracted and discretized with the preprocessor ANSA v18.1.0 (BETA CAE Systems, Farmington Hills, USA) by means of 97 820 fully integrated quadrilateral shell elements with a shell thickness of 0.03 mm. A mesh with elements distributed in rows along the longitudinal axis and a very fine taper‐meshing showed positive effects on the computations. The mesh was refined until no significant changes—ie, more than 1% of the maximum stress—was obtained. A further criterion for the mesh sensitivity of the balloon membrane was to find the ideal correlation between the number of shell elements along the smallest appearing radii and the computational time. Two hundred elements in the circumferential direction showed to be sufficient. The ESPRIT V1 stent was meshed with 119 680 fully integrated hexahedral elements. Here, three elements along the strut width and four through the thickness fulfilled the criteria for the mesh sensitivity. While the straight strut segments contained a coarse mesh, the connecting, bridge, and curved segments featured a symmetrical and fine element mesh. Only the contact surfaces of the blades of the folding, pleating, and crimping tools were implemented, as presented in Figure 5, and discretized with a coarse mesh. The folding blades' geometry is very complex because of their curved surfaces. The use of their correct dimensions is essential for realistic numerical results. Therefore, the blades were photographed frontally with a scale and their contours retraced with Autodesk Inventor 2018.

FEA simulations of the three preoperative processes and the stent deployment procedure were performed with LS‐DYNA (LSTC, Livermore, USA) with the implicit double precision version R10.1.0 for shared memory parallel processing. For the simulation times of the preoperative processes, the actual times of the respective work process were measured and adopted. The simulation time for the FEA of the CSI was adjusted according to the pressure ramp presented in Figure 4. Stresses and strains of the balloon membrane and the stent during the last time step of each simulation were transferred into the subsequent FEA. The stent and the balloon materials were based on a piecewise linear isotropic plasticity formulation in MAT24^49^ for the 316 LVM steel and MAT89^49^ for the Grilamid L25 polymer. The flow behavior of these models is defined by the von Mises flow rule:^50^

(1) $$\Phi =\frac{1}{2}s_{ij}s_{ij}-\frac{\sigma_{y}^{2}}{3}\leq 0,$$

where *s*_*ij*_ are the deviatoric stress components and *σ*_*y*_ is the yield stress, which is defined as

(2) $$\sigma_{y}=c(\sigma_{0}+f_{h}(\epsilon_{\text{eff}})).$$

The parameter *c* accounts for strain rate effects and scales the quasi‐static yield stress, *σ*_0_ is the initial yield strength, and *f*_*h*_ is a function of the effective plastic strain *ϵ*_eff_. For both material models, stress versus strain curves provided by the material manufacturers are implemented. In MAT24, the transition from the elastic to the plastic domain is characterized by the current yield stress. On the contrary, the material model MAT89 assumes the material to have yielded if the gradient of the user‐defined stress versus strain curve is smaller as the gradient defined by the Young modulus *E*. This method is well suited to approximately mimic the nonlinear behavior of polymers. However, the authors are aware that this model is not suitable to simulate the anisotropic material response of the blow‐molded balloon membrane. It can be expected that the alignment of the polymer chains in the circumferential direction allows the membrane to endure twice as much stress as in the longitudinal direction. To enable higher stresses within the ultimate tensile strength, the stress versus strain curves were extrapolated. Failure criteria to simulate the fatigue life of the balloon membrane were not defined because of the lack of data from biaxial tensile tests. Instead, the stress/strain curve in the plastic range was adjusted so that the inflation behavior of the balloon coincides as accurately as possible with the observations of the inflation tests. The inner and outer catheter shaft and the blades of all devices were defined as rigid. The blades were placed in a way that only a small gap of 0.01 mm between the balloon membrane and the stent was present. An artery model was not included, as this study mainly focuses on the influence of the preoperative processes on the simulation of the inflating balloon catheter and the expanding stent. For information about a constitutive model for wall mechanics of healthy and diseased arteries, we refer to, eg, Holzapfel et al^51^ with more references in there.

The nonlinear equilibrium equations are solved using the dynamic‐implicit Newmark‐*β* integration scheme,^37^, ^50^ firstly, because of the expected accuracy and, secondly, to account for the support situation of the system. In general, the equation system is solved for the time step *n*+1 as

(3) $${\text{Ma}}_{n+1}+{\text{Dv}}_{n+1}+\text{t}({\text{u}}_{n+1})=\text{f}({\text{u}}_{n+1}),$$

where **u** is the nodal displacement vector, **v** denotes the velocity vector, and **a** is the acceleration vector. Discretizing the weak form of the differential equation system yields the mass matri **M**, the vector of the internal reaction forces **t**, and the external forces **f**. The damping matrix **D** can be set up in several ways, eg, with constant or Rayleigh‐damping.

The Newmark‐*β* scheme introduces the following difference quotients:

(4) $${\text{u}}_{n+1}={\text{u}}_{n}+{\text{v}}_{n}\Delta t+\left[\left(\frac{1}{2}-\beta \right){\text{a}}_{n}+\beta {\text{a}}_{n+1}\right]\Delta t^{2},$$

(5) $${\text{v}}_{n+1}={\text{v}}_{n}+\left[\left(1-\gamma \right){\text{a}}_{n}+\gamma {\text{a}}_{n+1}\right]\Delta t,$$

which leads to an implicit difference equation in time. The parameters have been set to *β*=0.38 and *γ*=0.6, which introduce a certain amount of numerical damping. Because of the nonlinearity in the internal and external force vectors, a BFGS quasi‐Newton solution algorithm was applied.^50^, ^52^

LS‐DYNA allows controlling convergence of the solution by scaling the values for the convergence criteria with *ε*_d_,*ε*_e_, *ε*_r_, and*ε*_a_, which are the displacement, the energy, the residual, and the absolute tolerance, respectively.^53^, ^54^ After several runs, different criteria strategies were chosen. For folding and pleating, it was identified that efficient calculations could be performed if the displacement tolerance *ε*_d_ was loosened and set to

(6) $$\varepsilon_{d}=\left\|\Delta x_{k}\right\|/u_{\max}=0.01,$$

where $\left\|\Delta x_{k}\right\|$ is the norm of the incremental displacement, and *u*_max_ is the maximum attained displacement in any iteration *k*. To solve the more difficult problems of the stent crimping and the deployment process, *ε*_d_ and *ε*_e_ were disabled by putting them to large numbers. The residual tolerance*ε*_r_ was lowered to

(7) $$\varepsilon_{r}=\left\|F_{k}\right\|/\left\|F_{0}\right\|=0.01,$$

in which $\left\|F_{k}\right\|$ stands for the norm of the residual force and $\left\|F_{0}\right\|$ for the first residual vector for the implicit step *j*. In parallel, the absolute tolerance was set to*ε*_a_=−10 In LS‐DYNA, the negative value states that

(8) $$\left\|F_{k}\right\|<|\varepsilon_{a}|.$$

Contact treatment in implicit simulations of CSI is highly demanding. For this reason, penalty‐based, predefined segment‐to‐segment, automatic contacts based on the Mortar method^53^, ^54^, ^55^ were applied within LS‐DYNA. Although the friction model of Mortar contacts is based on the isotropic standard Coulomb law with a constant frictional coefficient only, it features options to overcome convergence problems and to increase the robustness and accuracy of implicit analyses. With large deformations of the balloon membrane, the normal vectors of the segments change permanently and thus also the contact parameters such as contact forces and segment velocity. This can cause convergence problems during implicit calculations if the balloon membrane and its contact partner—the blades and the stent—have different element sizes. With the Mortar method, an intermediate layer of contact elements is considered in which the node of each Mortar element is either a projection of a slave node or of a master node itself. In contrast to node‐to‐segment contacts, the calculation of the contact nodal forces is performed in this intermediate layer and follows the principle of virtual work. For the mathematical framework, the reader is referred to the work of Puso and Laursen.^55^ As a result, energy‐consistent contact stresses are calculated, which usually reduces convergence issues. The Mortar contacts were defined by part‐to‐part sets in which the rigid elements of the blades and shafts were generally chosen as master surfaces.

In the contact definition between the stent and the balloon membrane, the stent was classified as master and the much softer membrane as slave. In addition, single surface contacts for the stent and the balloon membrane had to be implemented. In all simulations, the outer nodes of the balloon membrane were attached to the rigid shafts via a node set and a constraint condition.

The rotary motion of every blade was defined by a pivot axis, represented by a vector, an angle *α*, and the process time mentioned above as presented in Figure 3. The respective axis of rotation was characterized by a unit vector **e** parallel to the longitudinal direction with its origin in a pivot point characterized by the position vector **p**. The endpoint **r** of **e** can then be expressed in matrix form as

(9) $$[\text{r}]=[\text{p}]+[\text{e}]=\left[\begin{array}{c} 0\\ \cos\left(\frac{2\pi }{n}i\right)R\\ \sin\left(\frac{2\pi }{n}i\right)R \end{array}\right]+\left[\begin{array}{c} 1\\ 0\\ 0 \end{array}\right],$$

where *n* stands for the total amount of blades of the respective process tool, *i* represents the number of the specific blade, and *R* is the radius from the tool center to the pitch circle of the pivot points.

In the folding and pleating processes, a ring of nodes of the membrane center was constrained in its longitudinal direction *x*. To enable elongation of the balloon catheter as close as possible to the motion inside the folding and pleating devices, both shafts were allowed to move and rotate along the *x*‐axis. Similar boundary conditions were used for the crimping process. The rotary motion of the balloon membrane was constrained around the *x*‐axis to support the wrapping mechanism of the wings. In a real stent expansion, both shafts allow certain movement along and around *x*. However, the authors decided to fully constrain the rotational degrees of freedom in *x* for both balloon ends. As a result, the angular momentum of the inflating wings of the balloon membrane and the expanding stent can be thoroughly analyzed.

For expanding the stent, the pressure on the inner surface of the balloon membrane was constantly increased to 1 MPa within 2 seconds according to the graph shown in Figure 4.

## 3 RESULTS

### 3.1 Folding process

The computational time for the folding process was 2 hours, 37 minutes on an Intel Core i7‐6700k CPU, 4.00 GHz, 32 GB (Intel Corporation, Santa Clara, USA) with a simulation time of 1 second and by using four cores. For the folding and the other processes, an automatic time step scaling with a maximum time step of 0.02 second was selected. The resulting star‐like cross section had a maximum diameter of *d*=1.77 mm.

**Figure 6:**
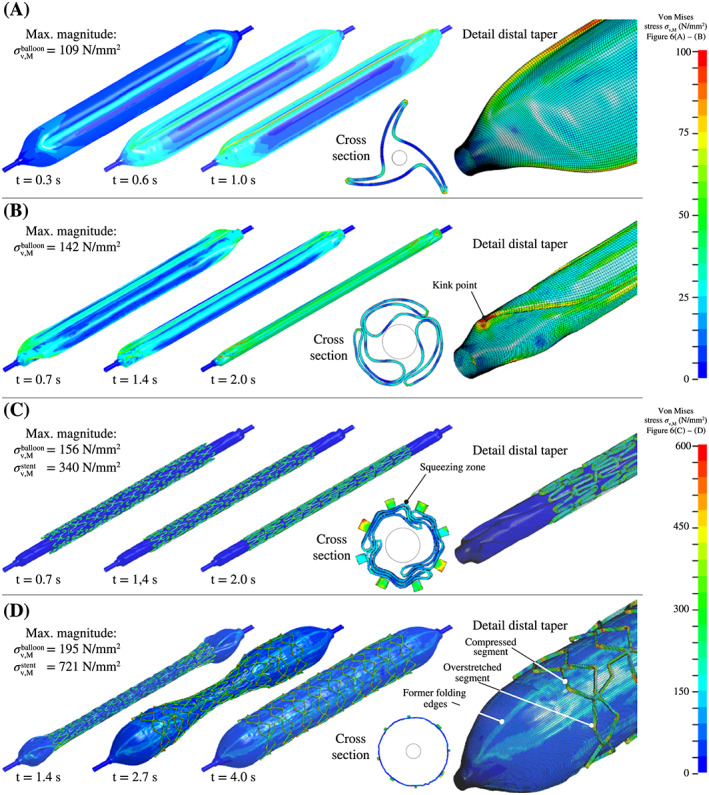
Distribution of the von Mises stress *σ*_v,*M*_ for the Baroonda stent delivery system (SDS) balloon catheter and the Esprit V1 stent during the preoperative processes A‐C, and the stent expansion D. The collection shows three isometric general views of the balloon and the stent after one‐ and two‐thirds of the simulation time and the final result. Furthermore, an out‐of‐scale sectional view taken from the center and a detailed view from the distal taper are presented. For better visualization, the blades of the respective devices of the preoperative processes are hidden. During the folding process (A), stress peaks occur at the outer wing edges of the balloon membrane. During the pleating process (B), the membrane gets heavily stressed, especially in kink points around the taper areas. In the crimping process (C), the membrane is additionally pressed into the gaps between the stent struts. Finally, during the expansion process (D), the floating stent segments, which lie between the membrane wings, get overstretched. As expected, stress peaks occur in the radii of the stent segments

As can be seen in Figure 6A, the balloon membrane experiences a significant deformation. Along the outer folding edges, extensive bruises could be detected with an overestimation in the von Mises stress distribution of more than 100 MPa. Despite the freely movable tapers, considerable buckling was obtained. During the procedure, the balloon lengthened by 0.5 mm. On the proximal side, the outer shaft rotated by 18.39°, and on the distal side, the inner shaft rotated by 19.43° around the *x*‐axis. This resulted in a twist of 1.04° between the proximal and distal ends. Because of the different diameters of the outer and inner shafts and thus a different shape of the proximal and distal tapers, an asymmetrical stress/strain field along the balloon membrane was determined. At the proximal end, ie, at the taper of the outer catheter shaft, significantly higher stresses were present. This is a result of the larger diameter of the distal end. Furthermore, in order to create the wings of the typical star shape, less membrane material needs to be deformed in order to obtain the same folding geometry.

### 3.2 Pleating process

**Figure 7:**
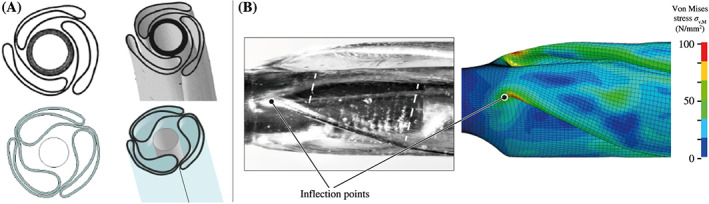
Comparison of the geometry of the folded and pleated Baroonda stent delivery system (SDS) balloon catheter taken from micro–computed tomography (CT) scans (A, top) and from the simulation (A, bottom). Inflection points can be detected in the light microscope images of the folded and pleated balloon and in the finite element analysis (FEA) (B). The positions of the inflection points change during pleating and move from the big to the small diameter of the tapers. This process causes streaks on the tapers (visible in the zone between the dashed lines)

From a numerical point of view, the most expensive task with a computational time of 14 hours, 27 minutes and a simulation time of 2 seconds was the pleating process. The geometries of all three wings show good agreement with the micro‐CT scans as presented in the comparison of Figure 7A. Large deformations of the balloon membrane required temporary small time steps.

During the proceeding compression of the three wings, inflection points appear along the folding edges in the outer areas of the tapers, causing high‐stress singularities with an overestimation of about 140 MPa, as shown in Figure 6B. These inflection points are also present on actual balloon catheters. Figure 7B displays a direct comparison. The positions of these inflection points moved from the big to the small balloon diameter until the process was finished. This seems to cause streaks on the surface, which can be again seen in the microscopic image of Figure 7B.

After finishing the simulation of the pleating process, it was verified that both tapers differed slightly in their shape because of the different shaft diameters. The final diameter *d* of the central cross section was 0.55 mm. The balloon catheter lengthened again by 0.14 mm, which resulted in a final length of the blank, folded, and pleated membrane of 27.16 mm. Also, a twist around the longitudinal direction of the proximal end of 58.72° and the distal end of 60.66° could be observed, which results in a twist of 2.94° during pleating and a final twist of 3.98° after the folding and pleating processes.

**Figure 8:**
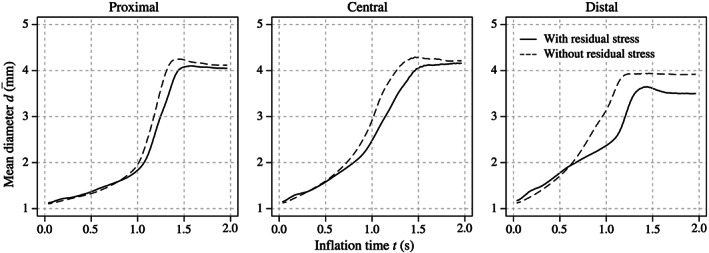
Simulation results showing the mean proximal, central, and distal diameter $\bar{d}$ of the Baroonda stent delivery system (SDS) balloon catheter during its inflation over time *t* of 2 seconds after incorporating or ignoring initial residual stresses. By ignoring residual stresses, it can be detected that all three sections of the balloon membrane expand faster and to a bigger end diameter

Figure 8 shows the inflation behavior of the balloon catheter by incorporating or ignoring initial residual stresses. Here, it should be mentioned that—apart from the implementation of the residual stresses—both simulation models were identical. To identify the differences in the expansion behavior, three diameters from the proximal, central, and distal sections of the balloon membrane were measured, from which each results from coordinates of three nodes lying on the tips of the folding edges of the wings. Afterward, the mean diameter $\bar{d}$ for every section was calculated. A comparison of the mean diameters indicates that in all three segments, the expansion of the membrane without initial residual stresses proceeded faster. Furthermore, differences in the final balloon diameters could be detected. The membrane without initial residual stresses continued to expand further, especially in the distal section.

### 3.3 Crimping process

The simulation of the last preoperative process—the crimping—with a computational time of 9 hours, 4 minutes and a simulation time of 2 seconds, was the second most expensive task—not including the FEA of the CSI. A high number of predefined contacts—a total of 15 contact segment‐to‐segment pairs and two single surface contacts—certainly had a strong influence on the computational effort.

Again, the stress in the balloon membrane increased to a maximum magnitude of 156 MPa. Parts of the membrane are squeezed into the gaps between stent struts, as presented in Figure 6C. A clear pattern of indenting stent struts was evident on the outer surface of the membrane. Furthermore, the balloon elongated by 0.016 mm and the stent by 0.1 mm. Because of the dodecagonal geometry of the crimping iris, segments of the stent get pressed into the corners formed by the blades, which resulted in a noncircular cross‐section of the crimped stent. In addition, the respective stent cells showed different compression ratios depending on their position. During crimping, the starting diameter of the untreated stent of *d*=1.94 mm got reduced to its smallest diameter of *d*=1.20 mm before it recoiled to its implantation diameter of *d*=1.28 mm.

### 3.4 Stent expansion

**Figure 9:**
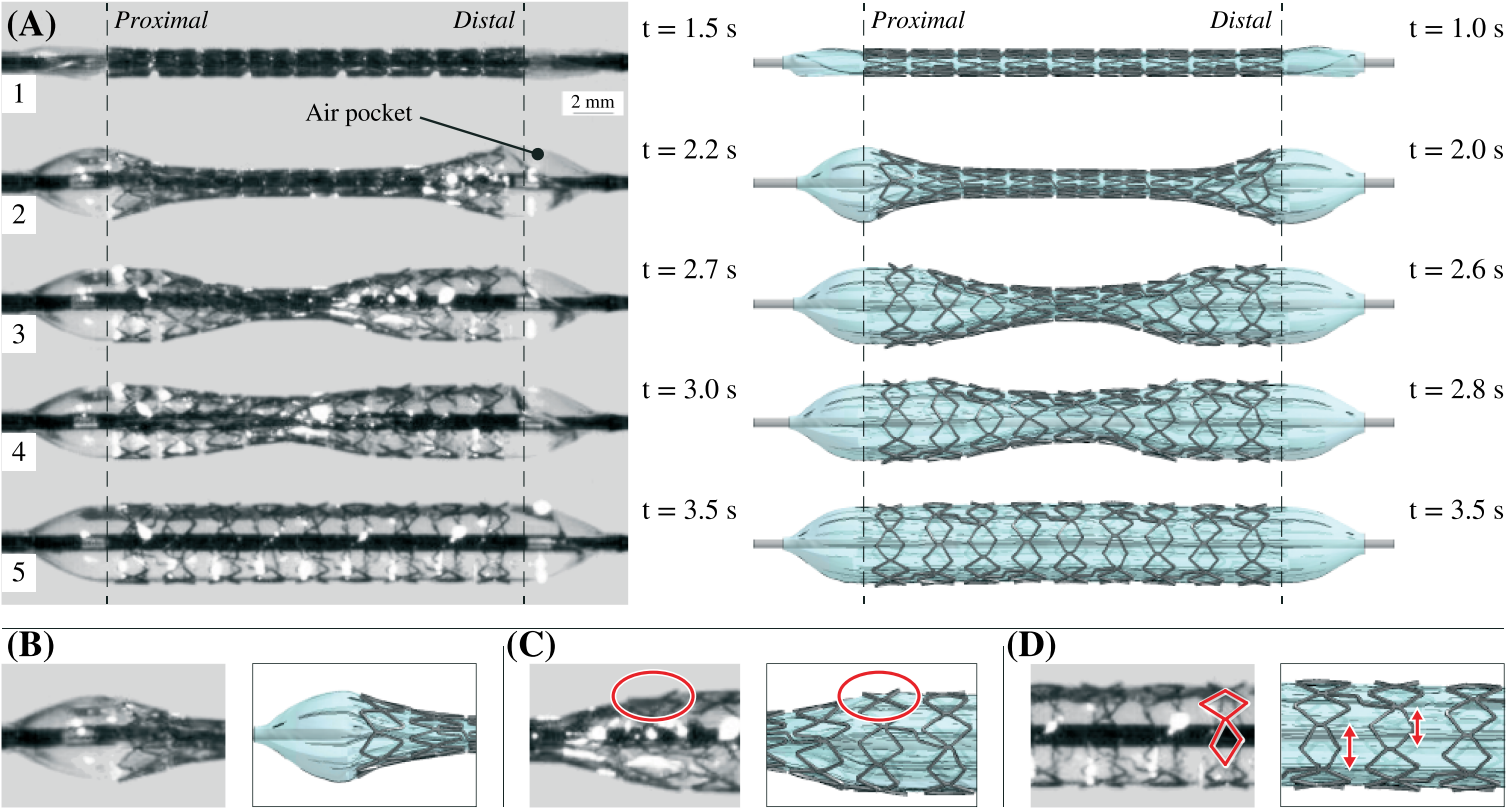
Expansive behavior of the stent delivery system (SDS). Overall, a very good agreement in the expansion behavior between the results of the experimental inflation test and the simulation can be observed A. As can clearly be seen, the stent first shortens and then slightly elongates. Furthermore, the presented finite element method shows that typical expansion mechanisms, such as dogboning B, the concave bending of single cells C, and the overexpansion of the cells located at the folding edges D, can be simulated

This last simulation was characterized by a computational time of 10 hours, 28 minutes and a simulation time of 4 seconds (Figure 6D). At about 1.3 seconds, the balloon and the stent started to show the typical dogboning effect (Figures 6D and 9A,B) in which the structurally weaker ends expand first. Towards the end of the simulation time, the stent rings expanded step by step from outside to inside. At the same time, the stent cells reached a maximum angle of 29.70° with respect to the longitudinal axis. Also, a slight tendency of the distal end to expand faster than the proximal end is recognizable in both the experimental and FEA images of the Figure 9A,B.

Because of dogboning and the resulting steep angle between the affected stent cells and the longitudinal axis, stent segments are bent outwards (Figure 9C). It is also noticeable that the cells, which are located at the folding edges, get overstretched by the lever of the expanding wings and thus show larger deformations and higher stress peaks (Figure 6D). Thus, a maximum stress of 721 MPa in one of the overstretched cells was achieved. During the expansion process, the stent first shortened from 19.6 to 18.0 mm at *t*=2.2 seconds before it elongated again to 18.5 mm at *t*=3.0 seconds. Because of the expansion mechanism of the balloon wings, the proximal end of the stent rotated 84.72° around the longitudinal axis and the distal end 93.05°. This results in a total twist of 8.33°.

**Figure 10:**
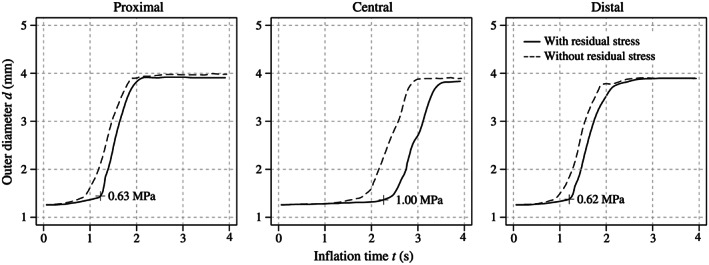
Simulation results showing the proximal, central, and distal diameter *d* of the Esprit V1 stent during its expansion after incorporating or ignoring initial residual stresses. By ignoring residual stresses, it can be detected that all three sections of the stent expand faster and in the proximal and central section also to a bigger final diameter. The burst point, ie, the beginning of the exponential increase of the diameter, is reached at a pressure of ∼0.60 MPa for the proximal and distal ends

Furthermore, two simulations—incorporating and ignoring residual stresses due to the preoperative processes—were performed to investigate the influence of previously experienced deformations of the balloon and the stent. As presented in Figure 10, the balloon and stent models incorporating residual stress expand faster and to a bigger diameter—although the latter applies only marginally.

**Figure 11:**
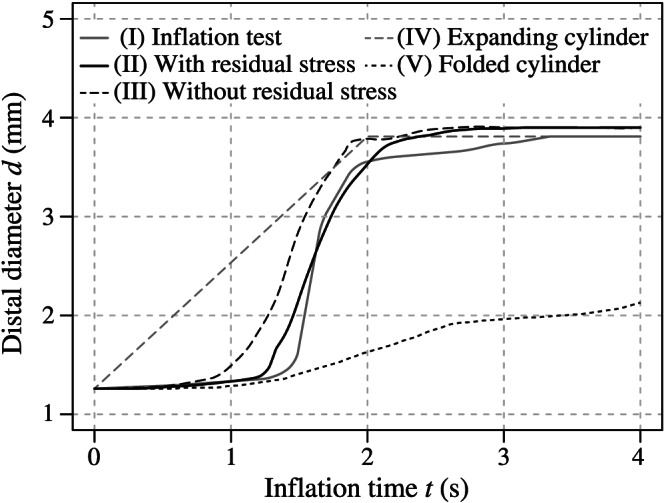
Distal stent diameter *d* against inflation time *t* of the experimental inflation tests (I) and of the finite element analysis (FEA) using the presented method incorporating initial residual stresses (II) and ignoring them (III) as well as of the FEA implementing an expanding cylinder (IV) and a folded cylinder (V) for the balloon model

Figure 11 presents the relationship between the outer diameter *d* of the distal stent end and the time *t* for the inflation test (I) and the FEA using the modeling method presented in this study (II) and classic approaches with the pure balloon geometry—which is taken from micro‐CT data or created because of the use of mathematical algorithm—without residual stresses (III) and implementing a displacement‐controlled expanding cylinder (IV) and a folded cylinder without tapers (V). Only the inflation test and the FEA using the presented method show a good agreement, especially if one considers that the expansion behavior of the balloon membrane depends on external influences such as the size of the air pockets inside the catheter, the viscosity of the expansion fluid, and the abrupt opening of the stent at a certain pressure. In the beginning, the proximal and distal stent diameter changes almost linearly, and, suddenly, it increases exponentially at a pressure of 0.62 MPa until it reaches its final size. On the contrary, the displacement‐controlled expanding cylinder is characterized by an expectedly linear expansion. Without the influence of faster expanding balloon tapers, the folded cylinder model expands slower and never reaches the final diameter within the simulation time.

**Figure 12:**
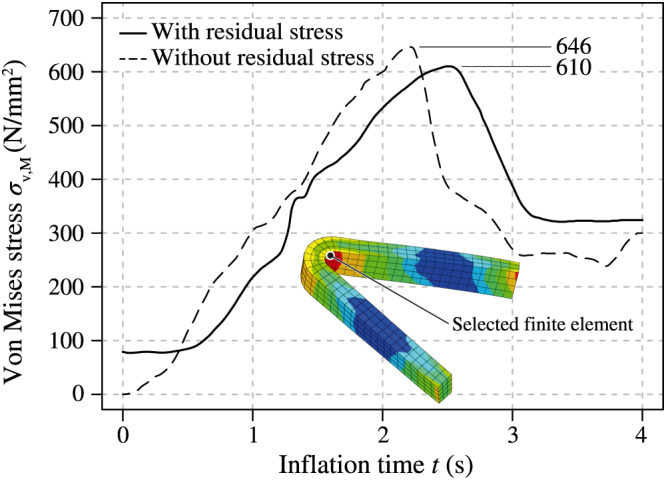
Von Mises stress *σ*_v,*M*_ vs inflation time *t* for the selected finite element incorporating and ignoring initial residual stresses due to the preoperative processes. The element is randomly taken from a stent cell located at the last ring of the distal stent end

When comparing the von Mises stress *σ*_v,*M*_ against the time *t* of the simulations with and without incorporating residual stresses, differences occur again, as shown in Figure 12. The maximum magnitude of the von Mises stress distribution in a curved segment of the outer ring of the distal stent end reached 610 MPa in the FEA with residual stresses and 646 MPa without residual stresses. Hence, the latter value almost exceeds the ultimate tensile strength of 671 MPa, which would incorrectly characterize the corresponding strut as a potential failure area.

## 4 DISCUSSION

This study presents a successful workflow for more accurate FEA of SDS and stent during CSI. The expansion behavior of the SDS, ie, the Baroonda SDS balloon catheter with its shafts and the ESPRIT V1 stent, showed in the simulation model a very satisfying agreement with the experimental results. Three factors contributed to this: (a) implementing detailed geometries of the balloon membrane and the stent without simplifying them; (b) incorporating initial residual stresses and strains experienced during folding, pleating, and crimping; and (c) using an implicit solver for the quasi‐static problem of a CSI.

To implement the detailed geometry of the stent and the balloon and their contact partners, the blades of the multihead tools of the preoperative processes was indispensable for a successful FEA. The production of a crimpable balloon catheter is a complex process in which the balloon membrane is pressed into the characteristic slanted star shape. In order to obtain a realistic cross‐section of the balloon, the implementation of the correct geometry of the folding blades was of great significance. The length and folding radii of the balloon wings depend on the folding blades. Also, the correct arrangement of the pleating and crimping blades according to the iris principle may influence the simulation results. Both the balloon membrane and the stent were pressed into the corners of the iris, creating a polygonal cross‐section. The methods known from the literature in regard to modeling the balloon geometry with the help of numerical, CAD, or imaging techniques,^33^, ^34^, ^35^, ^36^, ^37^, ^38^, ^39^, ^40^ and using a simple shrinking cylinder for crimping^41^, ^42^ instead of a pleating and a crimping iris seem not to be ideal for creating a realistic model for the FEA of the stent expansion.

Furthermore, a realistic geometry of the balloon membrane influenced the simulation of certain expansion mechanism of the stent. Since the tapers start to inflate first, they appear to be responsible for the opening impulse of the stent. At a certain pressure, the balloon wings in the area of the taper open explosively and thus push against the outer stent rings. The shortening of the balloon supported this mechanism. Figure 11 even indicates that a balloon without tapers is not able to start the stent expansion, even if the same inner pressure is applied. In addition, the tapers prevented the stent from showing abnormal dogboning and overexpansion. An indicator of this is the slightly bulgy shape that the balloon adopted during its expansion. Figure 8 proves that the maximum diameter is reached in the center of the balloon. This behavior cannot be precisely captured by using balloon catheter models without tapers, as known from the study of De Beule et al.^33^ As the results of this study demonstrate, both balloon and stent also twisted during the preoperative processes. A consistent strut width along the entire stent also favors pronounced dogboning. Individual cells can bend concavely (Figure 9C), which can cause stress peaks in the arterial wall and, therefore, severe vascular injuries. The authors believe that this effect could be prevented if excessive dogboning is counteracted by the reinforcement of the outer stent rings.

Furthermore , it has been shown that especially the tapers of the balloon are exposed to high stresses. Thus, the balloon membrane could already be significantly weakened during folding and pleating in addition to the damage described by Rondeau et al^45^ caused by crimping. Balloon tapers could typify the weak point of an SDS, which is why special attention must be paid to them during the early stage of development. However, the maxima in the von Mises stress distribution of the balloon membrane during the preoperative processes are an overestimation as they exceed the ultimate tensile strength. This is partly due to stress singularities, which occur at the folding edges and the inflection points. On the other hand, no failure criteria were defined in the material model, and the material curves were simply fitted to the experimental data. Therefore, to show the correctness of this finding, a new material model for the balloon membrane should be implemented as a future refinement step. This model must be able to mimic the anisotropic material response as well as the fatigue life of the membrane. The modeling approach must be based on data from biaxial tensile tests.

The use of a folded and pleated balloon model for the FEA of the stent expansion is crucial for the observation of expansion mechanisms of the whole SDS. As illustrated in Figure 9A, the cells of every ring did not open symmetrically. The reason for this mechanism is the partial lack of contact between some stent cells and the balloon membrane. This is particularly the case when the specific stent cell is located next to the folding edge of a balloon wing. When opening the wings, the cell loses contact with the membrane. Because of the lack of friction, this cell gets now overstretched. Elsewhere along the circumferential direction, friction prevents overstretching of cells. As a result, on the one hand, stent cells are exposed to different loads and experience nonuniform strains in the circumferential direction. Perhaps this can be prevented by developing new folding techniques, which generate balloons with symmetrical cross‐sections. On the other hand, the extent of stent cell deformation directly affects the shortening and elongation behavior of every individual stent ring and, consequently, of the whole stent in the longitudinal direction. Thus, the shear stresses induced in the arterial wall may also be influenced indirectly by the geometry of the implemented balloon and the stent models. This unsymmetrical cell mechanism cannot be detected in FEA studies, in which simple cylindrical balloon models have been implemented.^26^, ^27^, ^28^, ^29^, ^30^, ^31^ Therefore, the present approach seems to be superior.

The authors also advise against using only a quarter or half of the stent geometry, even if the stent is perfectly symmetrical along its longitudinal axis. Necessary degrees of freedom would be erroneously constrained. The FEA of the stent expansion disclosed that the stent end twist towards each other because of the influence of the different diameters of the balloon ends. Of course, the effect of this twist and all expansion mechanisms depend on the specific balloon and stent types. However, the authors are not aware of a balloon or a stent which feature an symmetrical geometry.

There is no question that a detailed geometry of an SDS can also be obtained from image data of micro‐CT scans. This could be shown by Mortier et al^38^ and by the scans presented in Figure 7. However, the micro‐CT method has some disadvantages, which is why the methodology of the present study is preferable. Firstly, micro‐CT scans are usually associated with high costs. This includes high acquisition or operating costs and also the license costs for the image processing software. Second, the processing of the image data is very time consuming. Thus, the scan of the SDS and the stent used in this study may take several hours. After that, the images must be preprocessed and segmented before the geometry can be cleaned, idealized, and meshed. Third, scans of balloon catheters and stents are often associated with a high number of disruptive image artifacts. The reason for this is the contrast ratio between the balloon membrane and the metallic stent. In addition, artifacts are induced by the X‐ray marker attached to the catheter.

Evidence of residual stresses and strains that stents experience during preoperative processes (balloon folding, pleating, and stent crimping) was already shown by Möller et al.^44^ Now, this study demonstrates that initial residual stresses and strains have a significant impact on the results of FEA of CSI. The expansion mechanism, the final geometry, and the stress distribution of the inflating SDS can be affected and, therefore, influence the optimization process of stents and balloon catheters. As presented in Figures 8 and 10, the balloon and the stent inflate faster and even overexpand to an unrealistic diameter if residual stresses are not implemented. This contradicts the non‐compliant nature of the membrane of the Baroonda balloon catheter. Also, the time interval between the full expansion of the stent ends and the central segments was smaller without residual stresses. This affected the extent of the dogbone and of the opening of the cells. As mentioned above, Figure 12 demonstrates that residual stresses prevented the stent from overexpansion. The stresses in the respective stent‐strut do not exceed the ultimate tensile strength. In this case, it seems that there is no need for further optimizations of the strut geometry, in contrast to the case when residual stresses are not considered. Therefore, the consideration of residual stresses and strains can significantly influence the factors time and costs during the optimization process of a stent. In addition, if a model of an artery is implemented in the FEA, the resulting changes in the kinematic energy of the expanding stent and the contact pressure between the stent and the arterial wall can falsify the resulting stress distribution inside the biological tissue.

**Figure 13:**
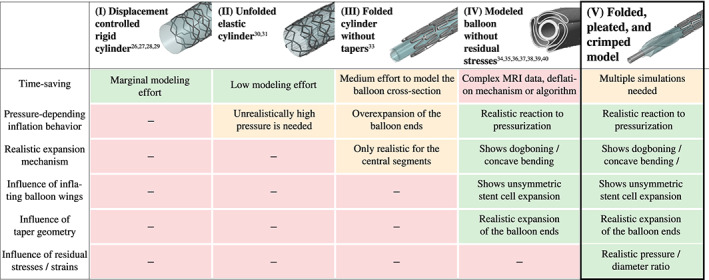
Comparison of all modeling approaches for balloon catheters for coronary stent implantation (CSI) simulations known from the literature: (I) displacement controlled rigid cylinder as balloon model^26^, ^27^, ^28^, ^29^; (II) unfolded elastic cylinder^30^, ^31^; (III) folded cylinder without tapers^33^; (IV) balloon model without residual stresses with a geometry gained from MRI data, by applying modeling algorithms, or by pressurizing the outer surface of the balloon^34^, ^35^, ^36^, ^37^, ^38^, ^39^, ^40^; and (V) folded, pleated, and crimped model—present approach. The refinement level is clearly increasing from the left to the right

Initial residual stresses also influenced the stress distribution of the balloon and the stent during the expansion of the SDS, as illustrated in Figure 12. It is noticeable that the implementation of the residual stresses resulted in lower stress magnitudes, but a higher stress concentration appears when the final stent diameter was reached. This supports the hypothesis of Schiavone et al^43^ which states that residual stresses may contribute to the flexibility of a stent during its deployment. Therefore, it can be assumed that a realistic geometry obtained from micro‐CT data or by mathematical algorithms is not sufficient to realistically simulate the stress/strain field of an SDS during the expansion phase. Figure 13 summarizes and underlines again the benefits of the presented method in comparison with the abovementioned refinement steps in the balloon catheter and stent modeling.

**Figure 14:**
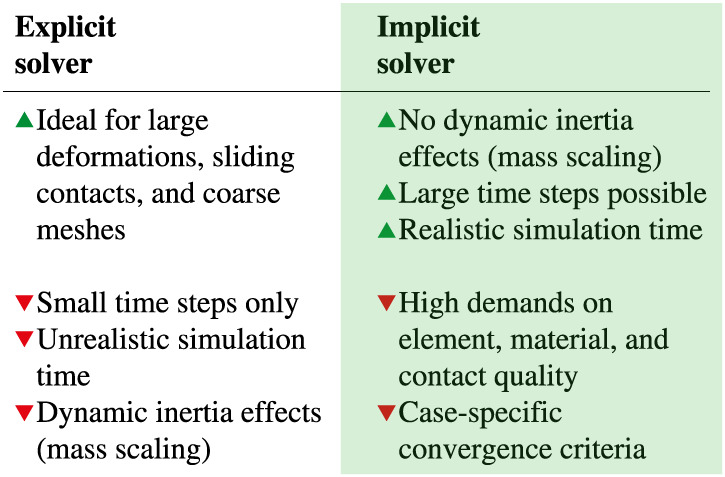
Comparison between explicit and implicit solvers. For solving the quasi‐static problem of a coronary stent implantation (CSI) in finite element analysis (FEA), the implicit solvers seem to offer more benefits

The implicit solver used for all performed simulations turned out to be very robust. Only minor modifications on the convergence criteria had to be undertaken. The authors believe that by eliminating dynamic inertia effects induced by mass scaling during explicit time integration, the accuracy of the simulations is increased. As implicit analyses have no inherent limit on increments of the calculations, larger time steps can be performed. This significantly decreases the computational time of all performed FEA. Furthermore, realistic process times could be simulated. These findings coincide with those of Bukala et al.^37^ Figure 14 shows the advantages and drawbacks of the implicit solver in comparison with the explicit method. For solving the quasi‐static problem of a CSI in FEA, the implicit solvers seems to offer more benefits.

Although the suggested method seems to be sophisticated, small time steps need to be chosen to enforce equilibrium for some problems. This included the intensive deformation of the balloon membrane during the folding process and the recoiling of the stent after crimping. The chosen parameters for the convergence criteria may not function for every problem. Here, different strategies must be deliberated and tested in a few trial iterations. Finally, special care must be taken in the contact treatment to establish contact between all parts during the initial time step.

Only if basically all relevant factors of CSI are considered, ie, the blood flow and the catheter injection medium, more precise material models, detailed geometries, and residual stresses and strains of all preoperative/interoperative and postoperative processes of the balloon membrane and the stent, a realistic load case for the artery can be simulated. This is the key for successful optimization processes of stents and other vascular implants such as stent grafts and transcatheter aortic valve implants. Therefore, and to continue the improvement of the presented method, the authors consider additional aspects for future studies. Balloon catheters usually tend to expand from their proximal to their distal end. Responsible for this is the fluid, which is injected into the catheter. It breaks through the cavities of the balloon membrane, which are separated by the stent segments. By adding more fluid, the internal pressure increases, the cavities get connected, and the stent rings expand step by step. This procedure could be simulated using the method of fluid‐structure interaction or by expanding the balloon catheter via the discrete element method. With such a model, a more realistic pressure/time behavior may be achieved. Own preliminary studies were already presented by Wiesent et al.^56^

Furthermore, an anisotropic material model of the balloon membrane with failure criteria needs to be implemented. Balloons of catheters are manufactured by blow molding. It is very likely the case that because of the production process, the polymer chains are orientated in the circumferential direction. In the future, thermomechanical effects should also be investigated, since the blades of the folding and pleating devices are heated to prevent the recoiling of the balloon membrane. Even though this is beyond the scope of the present study, in future investigations, the most important contact partner of a stent should be implemented—the artery. Therefore, a precise damage and growth model for the specific target vessel, based on experimental data, is needed.

In conclusion, the importance of preoperative processes of SDS and stents—balloon folding, pleating, and stent crimping—on the FEA of stent expansion models have been analyzed. The results demonstrate that the incorporation of initial residual stresses experienced during the previous production step significantly increased the accuracy of subsequent simulations and especially of the FEA of the stent expansion. During the preoperative processes, stresses inside the membrane and stent material also reached a severe level. Therefore, it can not be excluded that an SDS or stents are already damaged before the actual surgery. The implementation of the realistic geometry of an SDS, in particular the balloon tapers, and of blades of the process devices improved the simulation of important expansion mechanism, like dogboning and concave bending or overexpansion of stent cells. Furthermore, this study showed that up‐to‐date implicit solvers are able to precisely analyze all mentioned quasi‐static processes without the manipulation of the simulation time or the physical mass. Finally, the authors recommend to optimize the preoperative processes in a way that the resulting balloons catheters feature symmetrical cross‐sections and that stents show reduced dogboning and limited concave cell bending.
